# Effect of Chronic Oxidative Stress on Neuroinflammatory Response Mediated by CD4^+^T Cells in Neurodegenerative Diseases

**DOI:** 10.3389/fncel.2018.00114

**Published:** 2018-04-27

**Authors:** Helena Solleiro-Villavicencio, Selva Rivas-Arancibia

**Affiliations:** Laboratorio de Estrés Oxidativo y Plasticidad Cerebral, Departamento de Fisiología, Facultad de Medicina, Universidad Nacional Autónoma de Mexico, Ciudad de Mexico, Mexico

**Keywords:** oxidative stress, neurodegeneration, neuroinflammation, CD4^+^T cells, Treg cells, Th17 cells, Th2 cells, Th1 cells

## Abstract

In a state of oxidative stress, there is an increase of reactive species, which induce an altered intracellular signaling, leading to dysregulation of the inflammatory response. The inability of the antioxidant defense systems to modulate the proinflammatory response is key to the onset and progression of neurodegenerative diseases. The aim of this work is to review the effect of the state of oxidative stress on the loss of regulation of the inflammatory response on the microglia and astrocytes, the induction of different CD4^+^T cell populations in neuroinflammation, as well as its role in some neurodegenerative diseases. For this purpose, an intentional search of original articles, short communications, and reviews, was carried out in the following databases: PubMed, Scopus, and Google Scholar. The articles reviewed included the period from 1997 to 2017. With the evidence obtained, we conclude that the loss of redox balance induces alterations in the differentiation and number of CD4^+^T cell subpopulations, leading to an increase in Th1 and Th17 response. This contributes to the development of neuroinflammation as well as loss of the regulation of the inflammatory response in neurodegenerative diseases such as Alzheimer's (AD), Parkinson's (PD), and Multiple Sclerosis (MS). In contrast, regulatory T cells (Tregs) and Th2 modulate the inflammatory response of effect of T cells, microglia, and astrocytes. In this respect, it has been found that the mobilization of T cells with anti-inflammatory characteristics toward damaged regions of the CNS can provide neuroprotection and become a therapeutic strategy to control inflammatory processes in neurodegeneration.

## Introduction

Neurodegenerative diseases are characterized by the presence of a state of chronic oxidative stress and dysregulation of the inflammatory response. Wide evidence shows that persistent oxidative stress and neuroinflammation are key factors in the development and maintenance of the progressive neurodegeneration process in these diseases. The main characteristics of the state of oxidative stress are an increase of the levels of reactive species and a decrease in, or incapability, of the antioxidant systems to counter free radicals (Rivas-Arancibia et al., 2010; Halliwell, 2012).

In a redox balance state, the increase of free radicals and reactive species enhance transcription and synthesis of endogenous antioxidant defenses (Halliwell, 2012). These systems help to maintain the oxidation-reduction balance in the organism and play a restorative role during cellular damage. In this state, both the free radicals and reactive oxygen species (ROS) act as signals in several intracellular pathways, which are involved in cell metabolism and contribute to homeostasis maintenance (Rhee et al., 2003; Halliwell, 2012; Ray et al., 2012). However, during the chronic loss of redox homeostasis, ROS-induced intracellular signaling as pathways are altered, leading to a loss in the regulation of signals transduction by the cells. This occurs because the phosphorylation pathways are activated, while the dephosphorylation enzymes are inhibited. Therefore, the cells enter into a vicious cycle that cannot be broken (Rhee et al., 2003). In addition, when cell signaling is modified during oxidative state, the secretion of proinflammatory molecules is enhanced, and neoepitopes, as well as damage-associated molecular patterns, are produced. All these factors act together promoting a proinflammatory response (neuroinflammation in the central nervous system, CNS) (Bakunina et al., 2015).

The inflammatory response is modulated by oxidative stress. While in a redox balance the inflammatory response is a defense mechanism, which repairs, and is self-limited; in a chronic loss of redox balance—as it occurs in neurodegenerative diseases—the signaling pathways that modulate the immune system are altered, leading to the dysregulation of the immune response, which favors the predominance of pro-inflammatory responses (Griffiths, 2005; Jayaraj et al., 2017).

Besides the stimulation of ROS in the activation of the glia, several studies have demonstrated that CD4^+^T cells infiltrate into the CNS during the neurodegenerative process. They have also found that the contribution of these cells is highly important for the activation of the microglia and astrocytes (González and Pacheco, 2014; González et al., 2014).

In this review, we will explain the role of oxidative stress state on the loss of regulation of the neuroinflammatory response. This response is mediated through the effect of ROS on microglia, astrocytes, and CD4^+^T cells. Furthermore, we will describe the role that specific CD4^+^T cell subpopulations play in some of the most important neurodegenerative diseases.

## Oxidative stress and signaling

### Oxidative stress

Oxidative stress is defined as an imbalance between the pro-oxidant and antioxidant species, favoring the pro-oxidants. When chronic oxidative stress state is present leads to a disruption in redox signaling and cell damage (Halliwell, 2012). Oxidative stress is characterized by an increase in reactive species such as ROS and nitrogen reactive species (RNS). ROS include species such as superoxide anion (${\text{O}}_{2}^{•-}$), hydrogen peroxide (H_2_O_2_), and hydroxyl radical (^•^OH) (Halliwell, 2012; Ray et al., 2012). On the other hand, RNS include nitric oxide (NO) and peroxynitrite (ONOO^−^) (Halliwell, 2012; Ray et al., 2012).

Reactive species can be produced both endogenously and exogenously. Some enzymes in the cell organelles generate ROS and RNS endogenously; for example, the monoamine oxidase in the mitochondria; cytochrome P450 in the mitochondria, endoplasmic reticulum and plasma membrane; nitric oxide synthase (NOS) in peroxisomes; cyclooxygenases (COX) and nicotinamide dinucleotide phosphate oxidases (NOX) in cell membrane, and xanthine oxidase (XO) in the cytoplasm (Valko et al., 2007). On the other hand, the intracellular formation of free radicals can also occur by the influence of environmental factors such as ultraviolet radiation, ionizing radiation, and pollutants like ozone. Pro-oxidants can also have an external origin; some examples are environmental pollution, pesticides, heavy metals, some xenobiotics, and tobacco smoke. Reactive species *per se* and signals of cell damage -that derive from its action- can act as modulators of the inflammatory response.

### Reactive species as modulators of the inflammatory response

At physiological concentrations in the organism, ROS and RNS are regulators of several physiological functions. In a chronic state of oxidative stress, reactive species can become injurious, because they oxidize proteins and lipids, and they can damage DNA. Reactive species can also mediate the signaling that leads to the activation of the microglia and astrocytes (Pawate et al., 2004). At the same time, in diverse cellular populations, high concentrations of reactive species are able to activate signaling pathways and create vicious cycles that maintain a high secretion of proinflammatory cytokines and chemokines (Chan, 2001; Hsieh and Yang, 2013). Proinflammatory cytokines such as IL-6, IL-1β, tumor necrosis factor (TNF), and interferons (IFNs) induce the generation of ROS in non-phagocytic cells such as fibroblasts, vascular smooth muscle cells, endothelial cells, renal mesangial cells, and tubular cells.

NOX2 is a phagocytic enzyme that produces ROS in response to pathogens in a mechanism known as respiratory burst 10. The latter is a defense response of the organism that is accompanied by inflammation. However, this inflammatory response is self-limited and the redox balance is recovered after the pathogen has been eliminated. NOX2 has also been associated with the progression of inflammatory diseases such as atherosclerosis and pulmonary fibrosis, as it has been demonstrated that NOX2-deficient mice were protected against formation of atherosclerotic lesions and development of pulmonary fibrosis (Mittal et al., 2014). Furthermore, mice deficient in p47^phox^ subunit or NOX2 were also protected against TNF-α-induced lung inflammation or sepsis-induced lung microvascular injury (Manoury et al., 2005).

On the other hand, NOX of non-phagocytic cells (NOX4 and NOX5) have been recognized as an important source of ROS. Under physiological conditions, NOX derived from non-phagocytic cells show a constitutive activity of extremely low level. However, the enzymatic activity of this kind of NOX can increase in response to stimuli such as growth factors, cytokines, hyperglycemia, and hyperlipidemia, leading to the increase of the concentration of ROS (Meier et al., 1989; Bae et al., 1997; Li and Shah, 2003). NOX4 has been classically characterized as a kidney “oxygen sensor,” which regulates oxygen-dependent expression of erythropoietin and development of inflammatory processes in the kidney (Geiszt et al., 2000). Inflammatory stimuli such as LPS, TNF-α, hyperoxia, transforming growth factor beta (TGF-β), and hypoxia, were demonstrated to enhance ROS generation via NOX4 (Park et al., 2006; Basuroy et al., 2009; Ismail et al., 2009; Pendyala et al., 2009). For its part, the expression of NOX5 has been reported in human vascular endothelial cells and smooth muscle cells. NOX5 can be induced by thrombin (Belaiba et al., 2007), Ang II (Montezano et al., 2010), and platelet-derived growth factor (Jay et al., 2008). As well, an increased expression of NOX5 has been correlated with the oxidative damage observed in atherosclerosis (Guzik et al., 2008).

Another important source of ROS is the mitochondria. Mitochondrial-derived ROS results from the transfer of electrons across the mitochondrial membrane carriers. Particularly, ${\text{O}}_{2}^{•-}$ generated by mitochondria reacts with manganese superoxide dismutase (MnSOD) in the mitochondrial matrix to generate H_2_O_2_. When this molecule crosses the mitochondrial outer membrane to access cytosolic targets they can activate the redox-sensitive transcription factors (such as hypoxia-inducible factor 1 alpha, HIF-1α, and nuclear factor kappa beta, NF-κB) (Ungvari et al., 2007; Hamanaka and Chandel, 2009), upregulate the transcription of pro-inflammatory cytokines, and activation of inflammasomes (Naik and Dixit, 2011; Chen et al., 2015), all of them related to the inflammatory response.

Xanthine oxidase is the main superoxide-producing enzyme. The activity of XO is specifically induced by IFN-γ in lung microvascular endothelial cells and in the pulmonary artery endothelial cells. In this sense, XO-derived ROS have been associated with the development of a variety of inflammatory disorders such as ischemia-reperfusion injury, atherosclerosis, diabetes, and autoimmune disorders such as rheumatoid arthritis (Mittal et al., 2014). However, the therapeutic advantage of XO inhibitors such as allopurinol seems to be a promising approach in some of the inflammatory diseases that we have previously mentioned (Lee et al., 2009).

In addition, another important source of reactive species are the several receptors of growth factors that, when activated, stimulate the production of ROS. Among these are the epidermal growth factor receptor (EGFR) (Meng et al., 2007) and platelet-derived growth factor receptor (PDGFR) (Yang J. et al., 2016). Once the receptors are activated, they turn on the signaling pathways of protein kinases, which include tyrosine kinases, protein kinases C and mitogen-activated protein kinase (MAPK). As a consequence of the activation of these signaling pathways, cells synthesize and secrete proteins related to responses such as proliferation, differentiation and programmed cell death. The activation of these pathways is critical in the development of several pathologies. For example, the activation of MAPK p38 pathway contributes to neuroinflammation mediated by glial cells, including microglia and astrocytes (Chlan-Fourney et al., 2011).

**Figure 1:**
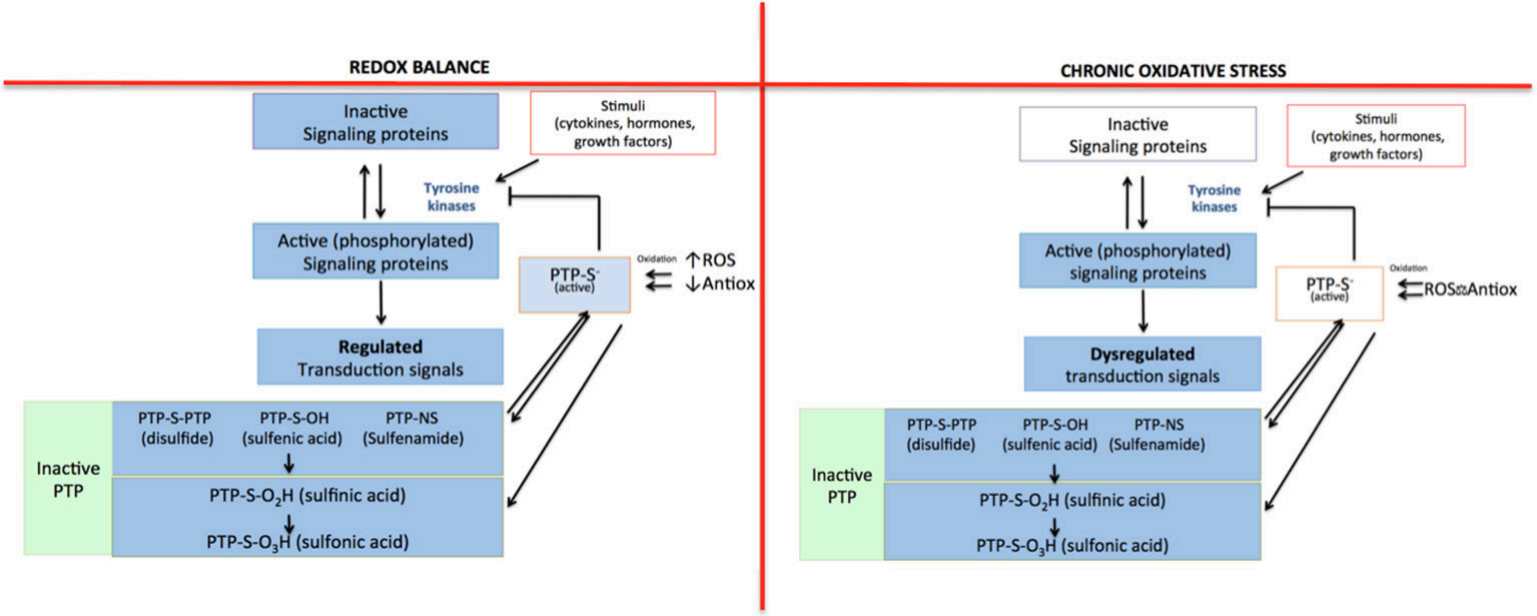
Indirect regulation mechanism for tyrosine kinases mediated by ROS. In redox equilibrium there is a balance between pro-oxidant and antioxidant species; for that reason, all the signaling pathways in which tyrosine kinases participate are regulated: protein tyrosine phosphatase (PTP) inhibits the activity of tyrosine kinases, limiting the signal. However, in a state of chronic oxidative stress, the loss of balance between pro-oxidant and antioxidant species causes an increase in ROS that inactivate PTP, which cannot inhibit the activity of tyrosine kinases. Then, the signaling pathway remains turned on, losing its regulation.

To this point, we have mentioned that reactive species are closely related to the inflammatory response that characterizes several diseases. They are able to induce the inflammatory response because they act as regulators of signaling pathways, as they are capable of oxidizing or reduce amino acid residues that are sensitive to redox changes (Corcoran and Cotter, 2013). The oxidizing modifications cause changes in the structure and/or function of proteins (Corcoran and Cotter, 2013). For instance, tyrosine kinases are one of the main protein families whose function is indirectly affected by the presence of ROS. The activity of this protein family is controlled by the action of the protein tyrosine phosphatases (PTP). However, ROS inactivate PTP by oxidizing cysteine residues in their catalytic site; therefore, tyrosine kinases remain active (Denu and Tanner, 2009; Cheng et al., 2011; Roos and Messens, 2011). Since there is no regulation of the activity of tyrosine kinases, signaling proteins within the pathway remain active (phosphorylated). In consequence, the signal transduction continues, leading to the expression of certain proteins that promote cell proliferation, apoptosis, and inflammatory response (Figure 1).

On the other hand, the alterations in the gene expression pattern are caused by the stimulation of ROS and RNS-sensitive regulatory transcription factors, which include nuclear factor E2-related factor 2(Nrf2), activator protein 1 (AP1), NF-kB, HIF-1α, p53, and Forkhead box O (FOXO). Many of these transcription factors have redox-sensitive cysteine residues in their DNA binding sites. At physiological levels, ROS and RNS induce the activation of the transcription factors mentioned above, and in this context, they effectively neutralize and remove the excess oxidants to restore redox homeostasis. Although, in an oxidative stress state, ROS and RNS over-stimulate transcription factors resulting in deficient cellular antioxidant defenses, generation of high levels of oxidative stress-inducing mediators, and alterations in signal transduction processes that lead to neurodegeneration (Farooqui, 2014).

## Neuroinflammation

### Characteristics of neuroinflammation

Inflammation is triggered to fight and control an injury, infection or other stimulus and it involves many cell types, as well as the secretion of soluble factors. In a redox balance, the inflammatory response is self-regulated, and is able to repair tissue damage and eliminate pathogenic elements. However, when the response is chronic, it causes an inflammatory environment that leads to progressive tissue damage (Inelia et al., 2016). Neuroinflammation is defined as the inflammatory response of CNS against elements that interfere with homeostasis. It is a combination of responses that involves the action of resident glial cells in CNS, which include microglia, oligodendrocytes, astrocytes, and non-glial resident myeloid cells (macrophages and dendritic cells) and peripheral leukocytes. This response is involved in all neurological diseases, such as traumatic, ischemic, metabolic, infectious, toxic, neoplastic, and neurodegenerative. Neuroinflammation plays an important role in the development and progression of neurological diseases. Thereby, it is important to understand and control the interactions between the immune system and the nervous system to prevent or delay the progression of CNS diseases.

Microglia and astrocytes are important contributors of neuroinflammation, thereby they play a major role in the progression of a wide variety of diseases, which are characterized by neuronal dysfunction and death. Other cell types including neurons, astrocytes, endothelial cells, etc., also express receptors for cytokines and other inflammatory mediators. These cells can be activated by the signals mentioned above, and participate in a coordinated inflammatory response in the brain. One of the common features of neuroinflammatory response in the neurodegenerative diseases, is the T cell infiltration into the CNS. It has been demonstrated in several neurodegeneration animal models, that T cell numbers increase, and their phenotype change during disease progression. However, in the human forms of these diseases, the dysregulation of the inflammatory response is difficult to study and thereby is not fully understood (Schettters et al., 2017). In the following sections, we will describe the role of microglia, astrocytes and CD4^+^T cells in neuroinflammation.

### Characteristics of neuroinflammation mediated by microglial and astrocytic cells

The microglial cells account for 10% of the total glial cells in human adult brain (Liu et al., 2015). The microglia of the CNS parenchyma consists of cells that are sensitive to changes occurring in their immediate environment (González-Scarano and Baltuch, 1999). These cells are the primary defense line against the presence of pathogen microorganisms and can detect critical changes in the activity and physiological state of neurons by interacting with them. In the healthy brain, neurons contribute maintaining microglia in a quiescent state by secreting factors that bind to receptors in the microglia membrane. Among them are the microglial receptors for the following molecules: the cluster of differentiation 200 (CD200), fractalkine, and some neurotransmitters such as γ-aminobutyric acid, acetylcholine, and noradrenaline (Korzhevskii and Kirik, 2016). An example of microglial regulation mediated by receptors is the binding of CD200 in neurons and CNS endothelial cells to its receptor CD200R, whose expression is predominant in myeloid cells, including macrophages and microglia. This is a mechanism to maintain microglia in a quiescent state (Hoek et al., 2000; Barclay et al., 2002).

Microglial cells can be activated by several molecules (Hanisch and Kettenmann, 2007), for example, matrix metalloproteinase 3 (MMP-3), α-synuclein, amyloid beta peptides (Aβ), neuromelanin and ATP. On the other hand, cell damage signals can also stimulate the microglia. Among these signals are heat shock proteins, calcium-binding proteins, proteases, uric acid, DNA, and high-mobility group box 1 protein (HMGB1). Additionally, the increase of ROS levels during the state of oxidative stress is a key factor to induce microglial activation. In this sense, it has been proven that exposure to ozone, which increases the state of oxidative stress in rats, leads to activation and phenotypical changes in the microglia (Rivas-Arancibia et al., 2010). Moreover, the stimuli that activate the microglia can bind to the receptors present in these cells (Doens and Fernández, 2014), including Toll-like receptors (TLRs), NOD-like receptors (NLR), the receptor for advanced glycation end products (RAGE) and scavenger receptors. In addition, the microglia has receptors for CD40 and CD91 ligands. All these receptors and their ligands contribute to microglial activation that leads to further production of cytokines and other inflammatory mediators, which may contribute to the apoptotic neurons death of in multiple neurodegenerative diseases (Owens et al., 2005). The most important cytokines that take part in the inflammatory process and that are secreted by the microglia are interferon gamma (IFN-γ), members of the TNF family, and several interleukins (IL-1β, IL-6, IL-8, and IL-12).

It has been described that microglial responses can have either a protective or a neurodegenerative effect, and this depends on the timing in which microglial activation appears in the disease. In an acute injury, microglial cells can cause oxidative and nitrative stress, but in this case, it is usually short-lived and helps to repair damaged tissue in the CNS. Although, when the stimulation is persisting for a long time, microglial cells can enter into an overactivated state in which the levels of ROS and RNS cause microenvironmental toxicity for surrounding neurons. Therefore, chronic microglial stimulation would trigger a chronic neuroinflammatory response, which is almost always harmful and damaging to nerve tissue (Krause and Müller, 2010).

On the other hand, astrocytes account for 20–30% of the total number of glial cells in the human brain (Pelvig et al., 2008). Under physiological conditions, astrocytes carry out numerous functions that are essential for neuronal survival (Bakunina et al., 2015); for example, the *crosstalk* between astrocytes and neurons, which occurs through the release of several neurotrophic factors (Choi et al., 2012). Besides, astrocytes are fundamental to the formation and regulation of the blood-brain barrier (BBB) (Hayashi et al., 1997).

As the microglia, astrocytes express several receptors; among them are the TLRs, which have usually been related to proinflammatory responses. Although, a study by Bsibsi et al. (2006) reported that, unlike the response of TLR3 observed in macrophages, the activation of the TLR3 signaling pathway in astrocytes is associated to the expression of anti-inflammatory cytokines such as IL-10. This cytokine promotes neuroprotection and the increase of neuronal survival. With respect to the capacity of astrocytes to release cytokines, chemokines and trophic factors, a study by Choi et al. (2014) investigated the secretome of human astrocytes by microarray. The study found that (unstimulated) cultured human astrocytes express eight pro-inflammatory factors: the granulocyte colony-stimulating factor (G-CSF), the granulocyte-macrophage-colony-stimulating factor (GM-CSF), chemokine ligand-1 (CXCL1), IL-6, IL-8, monocyte chemoattractant protein (CCL2), migration inhibitory factor (MIF), and serpin E1. This shows that astrocytes take part in the activation and chronicity of neuroinflammatory response. The activation of astrocytes is a critical factor in cell responses to brain injuries and chronic neurodegeneration (Pekny and Nilsson, 2005). The transition of astrocytes from a quiescent state to an active one has been observed in many neuroinflammatory conditions. The activation of astrocytes has been associated with the expression of the glial fibrillary acidic protein (GFAP); still, the molecular mechanisms underlying the induction of GFAP remain unclear (Pekny and Pekna, 2014).

Although the structural and communication functions of astrocytes have been well characterized, some evidence suggests that astrocytes also have the capacity of acting as immunocompetent cells (Dong and Benveniste, 2001). It has been observed that, under certain conditions as treatment with IFN-γ, astrocyte activation is characterized by an enhanced expression of the major histocompatibility complex (MHC)-II (Vardjan et al., 2012), which is a molecular complex implicated in antigen presentation. In addition, other studies revealed that astrocytes activated in culture with IFNγ, TNF-α, lymphotoxin, and IL-1 express CD80/86 (Soos et al., 1999) and CD40 (Abdel-Haq et al., 1999), which allow the costimulation signaling. The ability of astrocytes to express the molecules mentioned above, suggests that they can act similarly to professional antigen-presenting cells and, therefore, activate naive lymphocytes.

Due to their location and characteristics, astrocytes are in close contact with resident cells of CNS and blood vessels. The glial limiting membrane derived from astrocytes creates a barrier between the brain parenchyma and the vascular system (Ransom et al., 2003), known as the perivascular space, which is an accumulation site of immune cells in neuroinflammatory processes and is delimited by the endothelial basal membrane and the glia limitans (Bechmann et al., 2007). After the ligands bind to their respective receptor, astrocytes secrete chemoattractant factors that induce changes in the permeability of the BBB, leading to the recruitment and transmigration of leukocytes to the CNS. To enter the brain parenchyma, leukocytes need the secretion of MMP, which are able to break the glial limiting membrane (Rosell et al., 2008). This means that astrocytes play a critical role in the leukocyte infiltration into the CNS. The profile of leukocyte populations observed in neuroinflammation is highly selective. It is important to note that neutrophils—the most abundant leukocytes in the blood (70%)—are present in low concentrations within the CNS, as opposed to what occurs in inflammation in most of the tissues (Hickey, 2001). The main leukocytes observed in neuroinflammation are lymphocytes and mononuclear phagocytes, which constitute the most common populations in chronic inflammatory processes.

### The role of CD4^+^T lymphocytes in neuroinflammation

**Figure 2:**
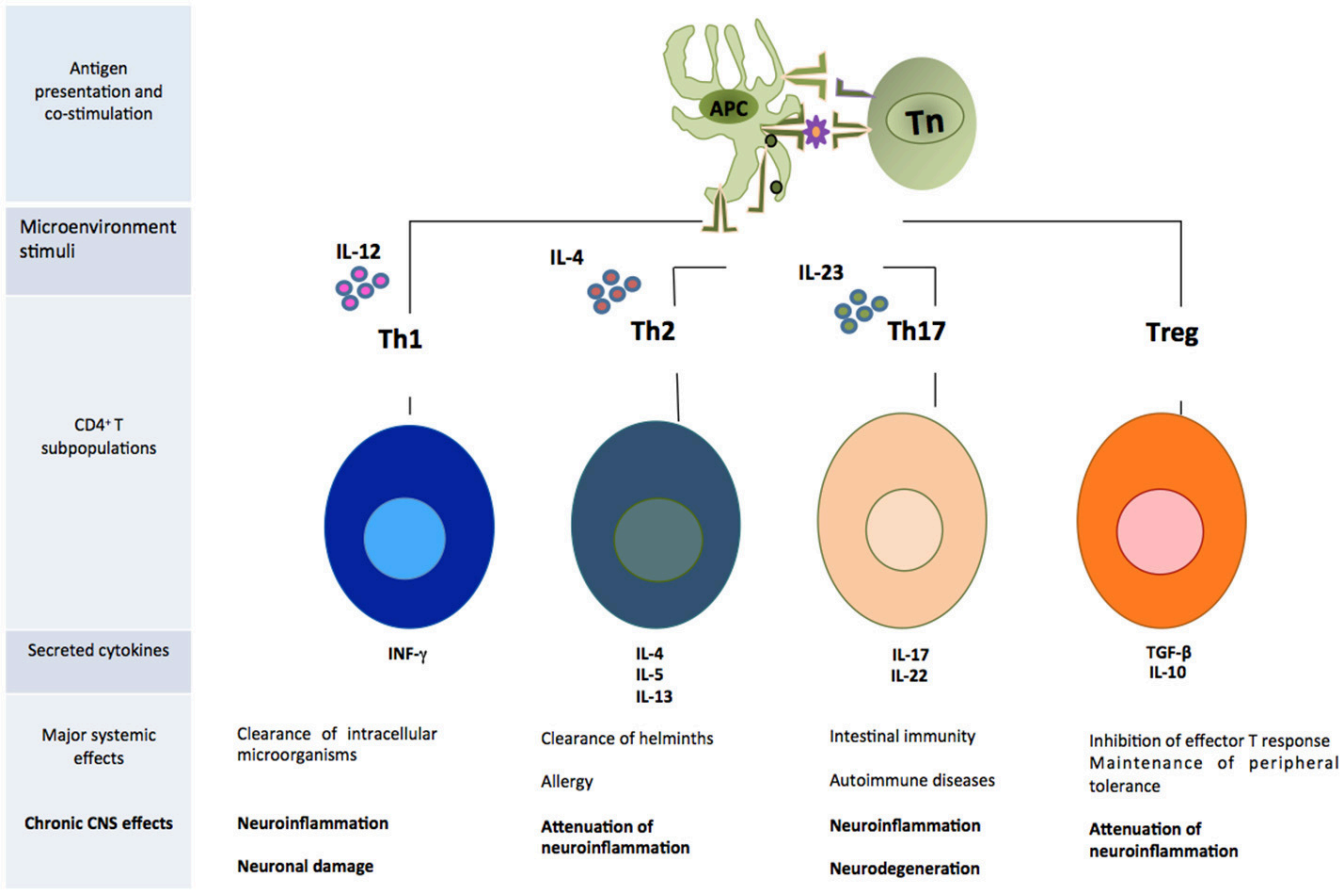
Subpopulations of CD4^+^T cells that play an important role in the development of neuroinflammation. APC, antigen presenting cell; Tn, T naive cell.

CD4^+^T cells are capable of activating and directing the functions of other cells. They participate in cellular mechanisms as antibody isotype switching and activation, and mobilization of cytotoxic T lymphocytes. They also regulate phagocytic and lytic activity of mononuclear phagocytes (microglial and tissue macrophages) (Male, 2014). Activated CD4^+^T cells can easily cross the BBB (Engelhardt and Ransohoff, 2005). Once they enter the damaged site, the cells exert several actions according to their phenotype (Vishal et al., 2012). Each cell subpopulation is specialized in coordinating immune responses against different types of threats and will produce particular effects. For example, in a medium where IL-12 is predominant, there will be a polarization toward the T helper 1 (Th1) phenotype, which has been associated to the elimination of intracellular microorganisms and causes neuroinflammation and neuronal damage in the CNS (Mosmann and Coffman, 1989). Th17 is another inflammatory phenotype whose differentiation is mediated by the presence of IL-23 (Bettelli et al., 2006). These cells participate in intestinal immunity, autoimmune diseases and have been related to neuroinflammation and neurodegeneration mediated by the activation of apoptotic Fas/FasL pathway (Zhang et al., 2008). On the other hand, differentiation toward Th2 phenotype occurs in a microenvironment where IL-4 is predominant. This cell subpopulation directs immune response against helminths and in allergy (Paul, 2010), but it is also involved in the attenuation of neuroinflammatory processes (Beers et al., 2008). Tregs are a cell subpopulation that suppresses the effector function of Th cells (Limón-Camacho et al., 2013). These cells usually participate in the maintenance of peripheral tolerance to own molecules, limiting inflammatory responses against exogenous antigens. Within the CNS they attenuate neuroinflammation and, in consequence, neurodegeneration (He and Balling, 2013) (Figure 2).

## CD4^+^T cells

### Regulation of CD4^+^T effector cells by oxidative stress

The equilibrium between oxidant and reducing species regulates the redox state in immunological cells. This is important since the changes in the concentration of reactive species are critical in the signaling of several biological mechanisms such as cell growth, apoptosis, and modulation of the immune response. Therefore, in pathological states in which there is a state of chronic inflammation, as in neurodegenerative diseases. The extended and persistent production of reactive species that exceeds the capacity of being countered by antioxidant systems, leads to a state of oxidative stress that affects the functions and differentiation of CD4^+^T cells. In general, it has been observed that the presence of ROS is required during redox balance for the adequate activation of T cells. *In vivo* studies have demonstrated that ROS act as a third activation signal upon T lymphocytes. This has been demonstrated with the evidence that the treatment with antioxidants, which reduces the amount of ROS, provides a deficient activation and proliferation of T cells of BALB/c, NOD, DO11.10, and BDC-2.5 mice (Hubert et al., 2007). Contrastingly, the increased concentration of ROS can lead to enhanced T-cell apoptosis, as a result of damage to the DNA and activation of genes induced by p53 and FasL (Kesarwani et al., 2013).

Subpopulations of T cells are characterized by the differential production of cytokines and cell distribution. They also show plasticity, that is, the ability to change from one lineage to another under certain inflammatory conditions (O'Shea and Paul, 2010) that can be affected by an oxidant microenvironment. In this respect, it has been proven that in an oxidative stress state, Th1 cells present an opposite response compared to Th2 cells (Frossi et al., 2008). In the study mentioned above, clones of Th1 and Th2 were used to evaluate the proliferation and secretion capability of cytokines as a response to oxidative stress. It was observed that low doses of H_2_O_2_ reduce the production of IFN-γ by activated Th1 cells, and increase IL-4 secretion by activated Th2 cells. It must be underlined that another study proved that T-cell activation by an oxidative signal originated in the mitochondrial respiratory complex I increases the expression of IL-2 and IL-4 (Kaminski et al., 2010). Moreover, using isolated T cells of atopic dermatitis patients, a study demonstrated that inhibition of ROS production mediated by complex I, blocks the hyperexpression of Th2 cytokines. Therefore, oxidative stress plays an important role by inhibiting the inflammatory response of Th2 phenotype. Similarly, another study documented the role of superoxide in T cell polarization (Hubert et al., 2010). In that study, NOX inhibition led to the production of superoxide in macrophages and T cells; as a result, the polarization of T cells was modified. The authors reported that, after activation with immobilized anti-CD3 and anti-CD28, NOX-deficient mice T cells showed an increase in Th17 phenotype while those cells with physiological levels of NOX produced cytokines related to Th1-like response. In addition, it has been observed that mitochondrial inhibitors of ROS, such as N-acetylcysteine and mitoquinone, lower differentiation to Th17 phenotype in a mouse model of IEX-1 gene deficiency (this deficiency increases apoptosis) (Zhi et al., 2012). Therefore, different levels of ROS exert a modulating effect upon T cells, both at activation and differentiation.

### Regulatory T cells and oxidative stress

Treg cells, compared to effector CD4^+^T cells, are less sensitive to oxidative stress-induced cell death, a fact that may be attributed to their observed high antioxidative capacity (Mougiakakos et al., 2011). This capacity is related to a higher expression of thioredoxins (Trx), which are ubiquitously expressed enzymes that facilitate cysteine thiol-disulfide exchange to reduce proteins. Trx counteracts oxidative stress by scavenging ROS, therefore they play an important antioxidant role in the organism. Tregs express and secrete higher levels of Trx-1 than effector CD4^+^T cells. It has been demonstrated that Trx-1 is critical for the resistance of Tregs to oxidative stress. At the same time, Trx-1 is responsive to inflammatory stimuli, such as TNF, a mechanism that enhances the survival of Tregs in an inflammatory millieu (Mougiakakos et al., 2011).

Regarding the effect of oxidative stress on Treg cells, results have been variable, depending on the type of study and disease. In this sense, it was recently discovered that high levels of free oxygen species target mitochondria and induce apoptosis in Treg cells, resulting in a suppressive cascade of Treg cells, as it occurs in the tumor microenvironment (Maj et al., 2017).

On the other hand, most of the studies agree that the state of oxidative stress exerts a negative influence on the function of Tregs. In order to explain this dysfunction in Tregs, several authors point out that it is related to the inhibition of Foxp3 expression. Firstly, under conditions of chronic oxidative stress there usually is an overproduction of IL-6, which inhibits Foxp3 expression during Treg differentiation (Yang P. et al., 2016). Oxidative stress can promote the enhanced production of nitric oxide, mitochondrial hyperpolarization, and Ca^2+^ influx. These are factors that promote the overexpression of the mechanistic target of rapamycin complex 1 (mTORC1), whose activity inhibits the proliferation of Tregs. However, mTORC1 promotes the expansion of Th1 and Th17 proinflammatory lymphocytes (Perl, 2016). Finally, leptin, a hormone primarily produced by adipocytes, also regulates T-cell differentiation. Leptin is an adipokine, whose increase has been associated with oxidative stress. It was recently found that leptin inhibits Treg proliferation and that its neutralization enhances TCR activity and expansion of Tregs (De Rosa et al., 2007; Margiotta et al., 2016). With this experimental evidence, we can establish that reactive species act as a third signal to take part in the differentiation and function of CD4^+^T cells. In a state of chronic oxidative stress, the excess of reactive species promotes differentiation toward proinflammatory phenotypes such as Th1 and Th17 and inhibits Tregs, which constitute an important peripheral mechanism of immune regulation.

## Oxidative stress, CD4^+^T cells, and neurodegenerative diseases

The pathogeneses of the neurodegenerative diseases are complex and there are not fully understood. As we have already mentioned, in physiological conditions, ROS derived from mitochondria, NOX, and XO are maintained at relatively low levels by the effect of antioxidants. However, redox balance can be disturbed by neural inflammation or abnormal mitochondrial function (Rego and Oliveira, 2003). In the neurodegenerative diseases, it is common to observe that aggregates of misfolded proteins and mitochondrial dysfunction are major inducers of ROS release.

**Figure 3:**
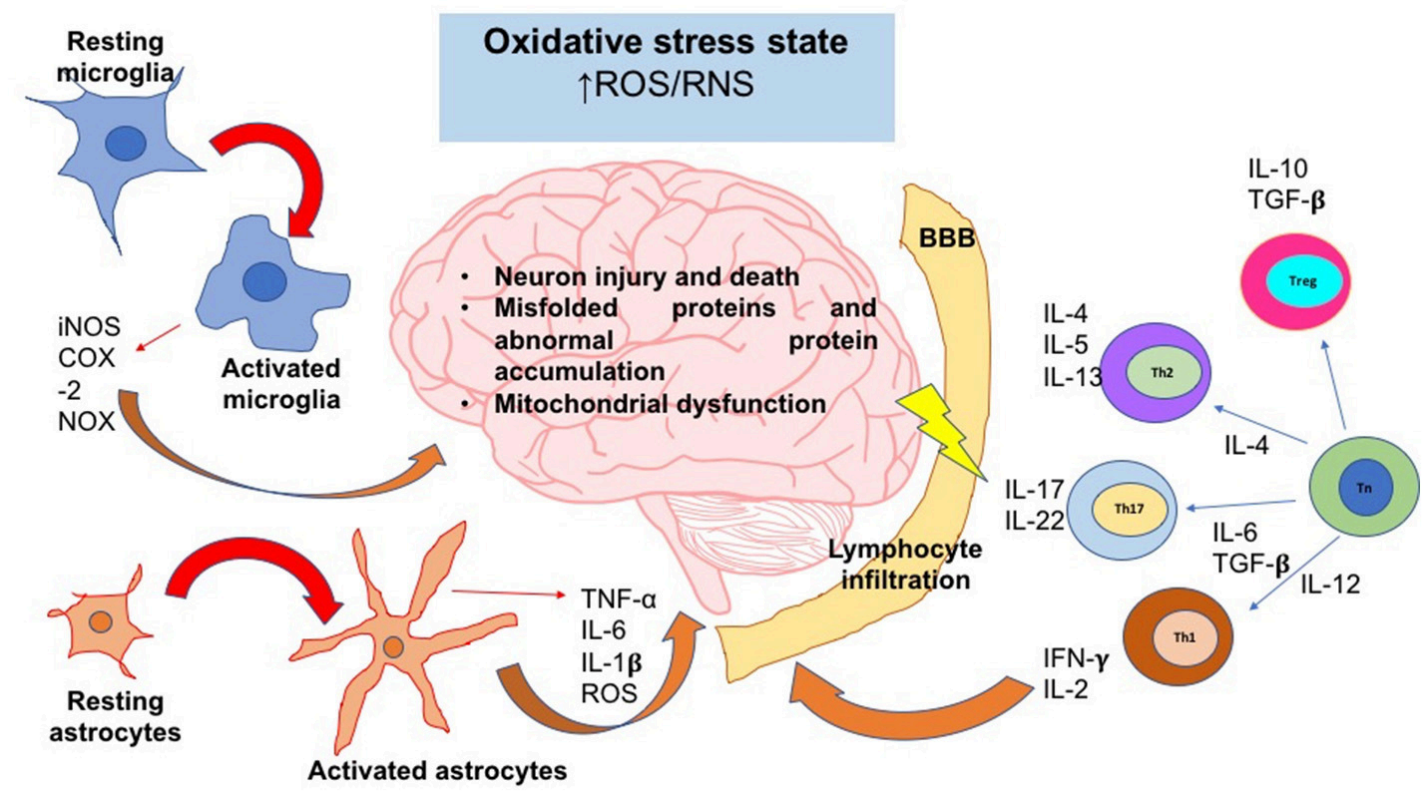
The oxidative stress state induces neuroinflammation and neurodegeneration. In an oxidative stress state, ROS and RNS levels are augmented; these reactive species can activate signaling pathways that lead to the activation of the major glial inflammatory characters: microglia and astrocytes. These glial cells secrete proinflammatory factors which positively feedback the neuroinflammatory response. On the other hand, SNC-secreted factors and peripheral cytokines are able to disrupt the blood brain barrier (BBB) integrity; thereby, leukocytes such as T cells are able to infiltrate into SNC and take turn in the positive feedback of neuroinflammation. Inflammatory cells and secreted factors lead to neurodegeneration, in which the most characteristic feature is the neuron injury and death. iNOS, inducible nitric oxide synthase; COX-2, cyclooxigenase-2; NOX, NADPH oxidase; IL, interleukin; Th, T helper cell; Tn, T naive cell; Treg, T regulatory cell; ROS, reactive oxygen species; RNS, reactive nitrogen species; TNF-α, tumor necrosis factor alpha; TGF-β, transforming growth factor beta.

We have previously reviewed that activation and differentiation of CD4^+^T cells, depends on the redox conditions of the microenvironment. These kinds of cells play a crucial role in the progression of neurodegenerative diseases; however, the intensity and type of CD4^+^T response vary between pathologies. In the next sections, we will describe the influence of oxidative stress in some of the most common neurodegenerative diseases, and the subpopulations of CD4^+^T cells are predominant under those conditions (Figure 3).

### Alzheimer's disease

Alzheimer's Disease (AD) is the most common neurodegenerative pathology worldwide. It is characterized by irreversible cognitive impairment and significant behavioral alterations (González and Pacheco, 2014). The pathophysiology of the AD is mainly associated with the extracellular deposition of Aβ plaques and the accumulation of intracellular tau neurofibrillary tangles (NFT) (Querfurth and LaFerla, 2010). Aβ 1-42 induces the release of calcium, from the endoplasmic reticulum to cytosol, where it accumulates. In consequence, endogenous levels of GSH decrease and ROS increase till they reach an oxidative stress state.

Oxidative stress has been identified as a key feature in the pathogenesis of AD, and has been associated with the deposition of Aβ. The Aβ plaques have been related to cellular effects, such as (1) the activation of p38 MAPK signaling pathway that leads to tau hyperphosphorylation, which, in turn, lead to intracellular NFT formation; (2) mediation of apoptotic pathways by triggering the death promoter Bcl-2, which leads to the mitochondrial release of cytochrome C, and 3) the infiltration of T cells into the brain parenchyma (Awasthi et al., 2005; Giraldo et al., 2014; Liu et al., 2017). On the other hand, CNS or systemic inflammation positively feedback ROS over-accumulation.

Regarding Th cell activity, there are reports of increased Th17 response in AD (Togo et al., 2002; Ferretti et al., 2016). In this sense, Saresella et al. (2011) conducted an immunophenotypic and functional analysis of T lymphocytes from peripheral blood of AD patients. The results obtained from this group were compared against those from subjects with mild cognitive impairment (MCI) and healthy controls (HC). In the study, the authors observed that, when stimulated *in vitro*, naive lymphocytes from AD patients increased the production of Th17-like cytokines (IL-21, IL-6, and IL-23) and expression of the RAR-related orphan receptor gamma (RORγt). Interestingly, there was a significant increase in the expression of the Th2-related transcription factor GATA-3 only in the group of MCI patients. These results suggest that the increase in GATA-3 observed in MCI patients might be a mechanism to modulate the activation of Th17 cells. The failure of this mechanism would allow the evolution of neuroinflammation that leads to the AD. In this respect, McQuillan et al. (2010) demonstrated that the production of IFN-γ and IL-17 by Th1 and Th17 cells, respectively, is inhibited by the transfer of Th2 cells. That is, the transfer of Th2 cells might have a regulatory and anti-inflammatory function in AD (McQuillan et al., 2010).

Regarding Tregs, some works using animal AD models have pointed out that these cells can critically regulate the response of Th cells, both in physiological and pathological environments (Baruch et al., 2015; Ye et al., 2016). A recent study conducted by Dansokho et al. (2016) analyzed the impact of Tregs on AD progression in a murine model of the disease. The authors found that the elimination of Tregs boosted the onset of signs of cognitive impairment in APPPS1 mice (mice in which the expression of human APP transgene is approximately 3-fold higher than in endogenous murine APP). On the contrary, when there was an amplification of the Treg population due to a peripheral injection of low doses of IL-2, the study reported an increase in the number of microglial cells, which helps to restore the cognitive functions in these mice. This work suggests that transfer of Tregs might have a beneficial effect on the regulation of the inflammatory response; this, in turn, might contribute to delaying the progression of the AD.

### Parkinson's disease

Parkinson's Disease (PD) is characterized by a 50–70% loss of dopaminergic neurons located in the substantia nigra (SN). The progressive degeneration of the dopaminergic fibers in the brain leads to the onset of prominent motor symptoms that characterize this disease (González and Pacheco, 2014). Several groups have provided evidence of the participation of oxidative stress and alterations in the immune system in the pathogeny of PD. In this sense, the over-accumulation of ROS or other free radicals has been related to the major pathological hallmark in PD: the degeneration of dopaminergic neurons90. As it occurs in the AD, excessive ROS production can be caused by mitochondrial dysfunction or inflammation. Mitochondrial dysfunction in PD has been related to complex I deficiency. Mutations in some target molecules have been associated with mitochondrial dysfunction, for example, PINK-1, DJ-1, parkin, α-synuclein and leucine-rich repeat kinase 2 (LRRK2). These mutations can impair mitochondrial function, leading to an increase of ROS levels and susceptibility to oxidative stress (Adam-Vizi, 2005; Zuo and Motherwell, 2013).

In regard to the molecular mechanisms involved in the death of dopaminergic neurons, a study by González et al. (2013) found that dopamine D3 receptors (D3R) expressed in CD4^+^T cells play a key role in the loss of dopaminergic neurons in SN. Using a PD animal model, an investigation found that when transferring CD4^+^T cells from wild-type (WT) mice to D3R-deficient mice, the latter became susceptible to neurodegeneration. On the other hand, RAG1^−/−^ mice (animals that lack T cells) are resistant to neurodegeneration induced by 1-methyl-4-phenyl-1,2,3,6-tetrahydropyridine (MPTP). However, these mice become susceptible to the loss of dopaminergic neurons when CD4^+^T cells from WT mice were transferred into them. The opposite occurs when the cells come from D3R-deficient mice given that these animals show no loss of dopaminergic neurons. The study also analyzed the activation and differentiation of CD4^+^T cells and found that the dopaminergic activation of D3R promotes T-cell activation and acquisition of Th1 phenotype. Therefore, the results of these work underline the importance of dopamine receptors in the activation of CD4^+^T cells and regulation of T cell-mediated immunity at *substancia nigra*, which contributes to neurodegeneration. Although these studies were carried out using an animal model, they suggest that D3R must be considered an important pharmacological target in PD treatment.

On the other hand, a study by Reynolds et al. (2010) observed that immunization with nitrated α-synuclein (N-α-syn) causes adaptive immune responses that exacerbate neuroinflammation and nigrostriatal degeneration in an MPTP-induced PD model. Such responses are primarily mediated by Th17 subset, which loses its regulation since there is a dysfunction of the Treg population. In PD, there is a chronic state of oxidative stress; hence, Tregs probably show hypoactivity and, in consequence, inability to regulate effector T cells. However, the administration of the vasoactive intestinal peptide (VIP), a known inductor of Treg activity, enhanced the neuroprotector capability of Tregs of mice treated with VIP in an MPTP-induced PD model. These results show that when administering a treatment that promotes the regulatory activity of Tregs, the neuroinflammatory response induced by N-α-syn can be modulated. The above provides the elements to continue developing and evaluating future vaccination or immunotherapy strategies using Tregs in PD.

### Multiple sclerosis

Multiple sclerosis (MS) is a chronic, progressive, demyelinating, and inflammatory disease (González and Pacheco, 2014). The loss of myelin is expressed in clinical symptoms as paralysis, muscle spasms, optic neuritis, and neuropathic pain. MS is characterized by destruction of myelin sheaths, axonal damage, the formation of a glial scar, and presence of inflammatory cells, primarily myelin antigen-specific Th cells (Kobelt et al., 2017).

In regard to oxidative stress in MS, most studies have related the lesion formation and progression to glial cells and recruited monocytes, which are the major producers of ROS. It has been demonstrated that ROS are critically involved in autoimmune-mediated tissue damage in MS. In this sense, observations in white matter and cerebral cortex lesions of MS autopsies suggest that demyelination and neurodegeneration could be a cause of the presence of oxidized lipids in myelin membranes, in apoptotic oligodendrocytes and in the axons of neurons (Haider et al., 2011; Fischer et al., 2013).

On the other hand, it is well known that cells belonging to the adaptive immune response, particularly Th cells play an important role in the pathogenesis of MS lesions. For decades, experimental autoimmune encephalomyelitis (EAE) has been the primary animal model of MS. It consists in inducing autoimmunity in mice after injecting peptides from myelin emulsified in adjuvant (Robinson et al., 2014). This model has also been applied to the study of the role of Th cells in MS. In the studies of adoptive transfer of EAE, researchers have proved that myelin-reactive Th1 or Th17 cells can trigger the disease in receptor mice; however, the histopathological results produced by the CD4^+^T cell populations are different from each other. In animals that received Th1 cells, the response of macrophages was more prominent, while Th17-cell mice showed a higher neutrophil infiltration (Kroenke et al., 2008; Fletcher et al., 2010). These differences are significant because, although neutrophils and macrophages are phagocytic cells, only macrophages are antigen-presenting cells. Then, they can activate cells of adaptive immunity, which promotes a chronic inflammatory response. Additionally, the findings previously described suggest that Th1 and Th17 cells have a prominent role in the development of lesions in MS given that both effector populations can cause inflammation in the CNS and demyelinating lesions; still, their effector mechanisms are different from each other.

It has been observed that encephalitogenic T cells in mice have multiple critical molecules that provide them the capability to induce inflammation in the CNS. For example, those Th1 cells that have been observed to have a high expression in integrin 4 (VLA4, a VCAM1 ligand present in endothelial cells) are encephalitogenic while those that have a reduced expression of VLA4 are not (Baron et al., 1993). As for the frequency of Tregs in MS, it has been observed that there are no significant differences in the number of these cells in peripheral blood or cerebrospinal fluid (CSF) in MS patients when compared against healthy controls. Nevertheless, the functional capability of Tregs is affected in MS patients (Fritzsching et al., 2011; Hsieh and Yang, 2013).

On the other hand, recent studies have proven that the elimination of Treg cells before EAE induction increases the acuteness of the disease (McGeachy et al., 2005; O'Connor and Anderton, 2008). This suggests that Tregs can suppress the expansion of autoreactive effector cells.

### Amyotrophic lateral sclerosis

Amyotrophic lateral sclerosis (ALS) is a neurodegenerative disease characterized by progressive degeneration of motor neurons in the cortex, brainstem, and spinal cord (National Institute of Neurological Disorders and Stroke, 2017). The term “amyotrophic” refers to muscular atrophy, and “lateral sclerosis” is related to the scarred aspect of the spinal cord (Malaspina et al., 2014). ALS is clinically characterized by paralysis, swallowing impairment and respiratory failure. Several forms of ALS have over 150 different mutations in SOD1 that include the entire genomic sequence, as well as the protein structure of SOD1 antioxidant system (Hooten et al., 2015). SOD1 is responsible for the conversion of ${\text{O}}_{2}^{•-}$ into H_2_O_2_ and O_2_, although SOD1 mutants enhance the production of Nox2-dependent ROS, inducing injury and death of motor neurons (Li et al., 2011). Oxidized or misfolded SOD1 has been shown to cause mitochondrial dysfunction, which contributes to the pathogenesis of spontaneous ALS (D'Amico et al., 2013). Motoneuron injury depends on the communication between motoneurons, surrounding glia and the immune system, which is enhanced in the oxidative stress state (Hooten et al., 2015).

The pathogenesis of ALS consists of 2 stages: the first one is an early neuroprotective stage in which the immune system is protective and restorative. In this stage, there is a predominance of M2-like macrophages and microglia, as well as a Th2 and Treg responses (Tiemessen et al., 2007; Beers et al., 2011). Interestingly, when Tregs are transferred into the ALS mouse (G93A mSOD1 transgenic mouse), the neuroprotective phase and survival were prolonged122. The progression of the disease is characterized by an increase in the motoneuron injury rate. Then, in the second phase, the macrophage and microglia's phenotype switches into M1. On the other hand, there is an infiltration of proinflammatory Th1 cells with the concomitant secretion of pro-inflammatory cytokines, such as IL-1, IL-6, TNF-α, and IFN-γ (Tiemessen et al., 2007; Beers et al., 2008). As we have previously mentioned, pro-inflammatory cytokines such as IL-6 can inhibit the phosphorylation of FoxP3, thereby reducing the suppressive function of Tregs. On the other hand, IL-1β promotes the conversion of Treg cells into IL-17-producing cells. The rapid progression of ALS is the result of the oxidative and pro-inflammatory milieu that positive feedbacks the differentiation and proliferation of Th1 and Th17 cells.

## Conclusion

The chronic state of oxidative stress induces cell damage and progressive neurodegeneration since the regulation of the inflammatory response is lost. This occurs because reactive species can act as signaling molecules. During the chronic state of oxidative stress, those reactive species promote the sustained activation of the signaling pathways, these lead to the expression of factors that in turn promote the deregulation of the inflammatory response.

In of the progressive neurodegeneration processes, in which there is a state of chronic oxidative stress, the increase in ROS induces an increase in proliferation and proinflammatory activity in astrocytes and microglia. Moreover, different studies show that redox balance is a key factor in processes such as activation, proliferation, and differentiation of T cells. Therefore, a state of oxidative stress promotes polarization toward proinflammatory phenotypes as Th17 while Treg activity is inhibited.

The modulation of the cell redox state is proposed as a therapeutic strategy with which the activation and differentiation of CD4^+^T cell subpopulations could be regulated. Furthermore, Treg cell transfer together with an antioxidant treatment must be considered a promising strategy in the treatment of neurodegenerative diseases.

## Author contributions

HS-V was the responsible for the search and revision of the bibliography that was used in this article. The figures and tables were designed by HS-V. The writing of this article was done by HS-V and SR-A. SR-A collaborated in the style correction of this work.

### Conflict of interest statement

The authors declare that the research was conducted in the absence of any commercial or financial relationships that could be construed as a potential conflict of interest.
